# AI-Driven Models for Diagnosing and Predicting Outcomes in Lung Cancer: A Systematic Review and Meta-Analysis

**DOI:** 10.3390/cancers16030674

**Published:** 2024-02-05

**Authors:** Mohammed Kanan, Hajar Alharbi, Nawaf Alotaibi, Lubna Almasuood, Shahad Aljoaid, Tuqa Alharbi, Leen Albraik, Wojod Alothman, Hadeel Aljohani, Aghnar Alzahrani, Sadeem Alqahtani, Razan Kalantan, Raghad Althomali, Maram Alameen, Ahdab Mufti

**Affiliations:** 1 Department of Clinical Pharmacy, King Fahad Medical City, Riyadh 12211, Saudi Arabia; 2 Department of Medicine, Gdansk Medical University, 80210 Gdansk, Poland; 3 Department of Clinical Pharmacy, Northern Border University, Rafha 73213, Saudi Arabia; 4 Department of Pharmacy, Qassim University, Buraydah 52571, Saudi Arabia; 5 Department of Medicine, University of Tabuk, Tabuk 47911, Saudi Arabia; 6 Department of Medicine, Qassim University, Buraydah 52571, Saudi Arabia; 7 Department of Medicine, Al-Faisal University, Riyadh 12385, Saudi Arabia; lsalbraik@alfaisal.edu; 8 Department of Medicine, Imam Abdulrahman Bin Faisal University, Dammam 31411, Saudi Arabia; 9 Department of Medicine and Surgery, King Abdulaziz University, Jeddah 22230, Saudi Arabia; hmaljohani@stu.kau.edu.sa (H.A.); rkalantan0006@stu.kau.edu.sa (R.K.); 10 Department of Medicine, Al-Baha University, Al Bahah 65964, Saudi Arabia; 11 Department of Pharmacy, King Khalid University, Abha 62217, Saudi Arabia; 12 Department of Medicine, Taif University, Taif 26311, Saudi Arabia; 13 Department of Medicine, Ibn Sina National College, Jeddah 22230, Saudi Arabia

**Keywords:** AI-driven models, diagnosing, predicting, outcomes, lung cancer, systematic review, meta-analysis

## Abstract

**Simple Summary:**

This research explores the transformative potential of artificial intelligence (AI) in the early detection of lung cancer. Through a comprehensive systematic review and meta-analysis, this study evaluates the effectiveness of AI models, emphasizing a promising avenue for improving diagnostic accuracy. Among 1024 identified records, 39 studies were meticulously selected and analyzed following the PRISMA guidelines. The findings highlight significant strides in AI’s role, emphasizing the need for standardized protocols. Despite the observed heterogeneity, this study underscores AI’s promising impact on lung cancer screening, laying the groundwork for future advancements in clinical practice. This research contributes crucial insights for healthcare professionals and researchers alike, aiming to enhance the early diagnosis and management of lung cancer.

**Abstract:**

(1) Background: Lung cancer’s high mortality due to late diagnosis highlights a need for early detection strategies. Artificial intelligence (AI) in healthcare, particularly for lung cancer, offers promise by analyzing medical data for early identification and personalized treatment. This systematic review evaluates AI’s performance in early lung cancer detection, analyzing its techniques, strengths, limitations, and comparative edge over traditional methods. (2) Methods: This systematic review and meta-analysis followed the PRISMA guidelines rigorously, outlining a comprehensive protocol and employing tailored search strategies across diverse databases. Two reviewers independently screened studies based on predefined criteria, ensuring the selection of high-quality data relevant to AI’s role in lung cancer detection. The extraction of key study details and performance metrics, followed by quality assessment, facilitated a robust analysis using R software (Version 4.3.0). The process, depicted via a PRISMA flow diagram, allowed for the meticulous evaluation and synthesis of the findings in this review. (3) Results: From 1024 records, 39 studies met the inclusion criteria, showcasing diverse AI model applications for lung cancer detection, emphasizing varying strengths among the studies. These findings underscore AI’s potential for early lung cancer diagnosis but highlight the need for standardization amidst study variations. The results demonstrate promising pooled sensitivity and specificity of 0.87, signifying AI’s accuracy in identifying true positives and negatives, despite the observed heterogeneity attributed to diverse study parameters. (4) Conclusions: AI demonstrates promise in early lung cancer detection, showing high accuracy levels in this systematic review. However, study variations underline the need for standardized protocols to fully leverage AI’s potential in revolutionizing early diagnosis, ultimately benefiting patients and healthcare professionals. As the field progresses, validated AI models from large-scale perspective studies will greatly benefit clinical practice and patient care in the future.

## 1. Introduction

Lung cancer remains a formidable global health challenge, claiming the lives of millions of individuals each year [1]. The high mortality rate associated with lung cancer is primarily attributed to the advanced stage at which it is often diagnosed [2]. Lung cancer is notorious for its asymptomatic early stages, making it extremely difficult to diagnose until it has reached an advanced, often incurable, stage. The later the diagnosis, the more limited the treatment options, and the grimmer the prognosis for patients. In contrast, when lung cancer is detected at an early stage, the chances of successful treatment and long-term survival increase significantly. Consequently, there is a pressing need for innovative strategies to enable early detection, as this could significantly improve the prognosis and overall survival rates of lung cancer patients [3]. In recent years, the field of artificial intelligence (AI) has emerged as a promising avenue for achieving this goal. In the field of healthcare, AI has shown promise in improving diagnostic accuracy, predicting disease outcomes, and personalizing treatment plans [4]. In the context of lung cancer, AI systems can analyze vast datasets of medical images, patient records, and genetic information to identify patterns and abnormalities that may elude human perception. These systems can not only detect lung cancer at earlier stages, but also assist in risk assessment and treatment planning [5].

Current methods for early lung cancer detection include screening programs such as low-dose computed tomography (LDCT) and the analysis of biomarkers. While these approaches have demonstrated some success, they are not without limitations [6]. LDCT, for instance, may lead to overdiagnosis and increased healthcare costs. AI systems can potentially enhance the effectiveness of these methods by providing more precise and efficient analysis, reducing false positives and false negatives, and offering a complementary approach to existing techniques. Despite the potential benefits of AI in early lung cancer detection, several challenges and considerations must be addressed [7,8]. The performance of AI models can vary depending on the quality and diversity of the data used for training [9,10]. Therefore, the selection and curation of data are fundamental to the success of AI-based systems in this context.

This systematic review and metanalysis endeavors to provide a comprehensive evaluation of the performance of AI systems for the early detection of lung cancer. This paper analyzed the current state of AI applications in lung cancer detection, including the various techniques and approaches being utilized. Furthermore, the study critically assessed the advantages and limitations of AI-based methods compared to traditional approaches.

## 2. Materials and Methods

In conducting this systematic review and meta-analysis evaluating the performance of AI systems for the early detection of lung cancer, we meticulously adhered to the Preferred Reporting Items for Systematic Reviews and Meta-Analyses (PRISMA) 2020 guidelines. The following detailed Method Section outlines the steps taken in this comprehensive review.

### 2.1. Protocol Development

The research question was formulated to assess the performance of AI systems in early lung cancer detection. A detailed protocol was developed, outlining the inclusion and exclusion criteria, search strategy, and methods for data extraction and analysis. In accordance with the journal’s guidelines, this systematic review was not registered in any specific database prior to its initiation. While the journals encourage registration for systematic reviews, it is not a mandatory requirement universally practiced in the field. This decision was aligned with established practices within this domain, considering the extensive body of previously published systematic reviews without prior registration in reputable peer-reviewed journals.

### 2.2. Literature Search

Comprehensive searches were conducted in electronic databases, including PubMed, Google Scholar, Science direct, and Embase, to identify relevant studies published up to October 2023. The inclusion of Google Scholar helped us to identify the grey literature, conference papers, and other non-traditional sources of information.

### 2.3. Search Strategy

The search strategy included a combination of keywords and Medical Subject Heading (MeSH) terms related to lung cancer, artificial intelligence, and early detection. The search strategy was tailored to each database to account for variations in syntax and indexing. This ensured that no relevant studies were missed.

### 2.4. Study Selection

Two independent reviewers screened titles and abstracts for eligibility based on predefined criteria. The initial screening of titles and abstracts helped in rapidly identifying studies that met the inclusion criteria and eliminating those that did not. Full-text articles of potentially relevant studies were retrieved for further assessment. A full-text review was conducted on potentially relevant studies to ensure that the selection process was rigorous, and that the final dataset was of high quality.

### 2.5. Eligibility Criteria

#### 2.5.1. Inclusion Criteria

Studies evaluating the performance of AI systems for the early detection of lung cancer.

Original research articles published in English.

Studies reporting sensitivity, specificity, and other relevant diagnostic performance metrics.

#### 2.5.2. Exclusion Criteria

Studies lacking sufficient data on AI system performance.

Reviews, commentaries, and conference abstracts without primary data.

### 2.6. Data Extraction

Relevant data, including study characteristics, AI system details, validation methods, and diagnostic performance metrics, were extracted using a standardized data extraction form. Two reviewers independently extracted data from selected studies. Discrepancies were resolved through discussion or consultation with a third reviewer.

### 2.7. Quality Assessment

The quality of the included studies was assessed using appropriate tools, considering factors such as study design, patient selection, and AI system evaluation. A risk-of-bias graph and summary were generated to visually represent the methodological quality of the included studies.

### 2.8. Data Synthesis and Analysis

The meta-analysis was carried out using R software (Version 4.3.0, Vienna, Austria) along with the RStudio interface (Version 2023.03.0, Boston, MA, USA). Packages and libraries such as meta and metafor were utilized to calculate key performance metrics, including pooled sensitivity and specificity, along with their associated confidence intervals, i.e., 95%. Moreover, the presence of heterogeneity among the included studies was evaluated using a chi-square test and I^2^ index statistics.

### 2.9. Reporting

A PRISMA flow diagram was used to illustrate the study selection process, including the number of studies identified, screened, assessed for eligibility, and included in the final analysis.

## 3. Results

**Figure 1:**
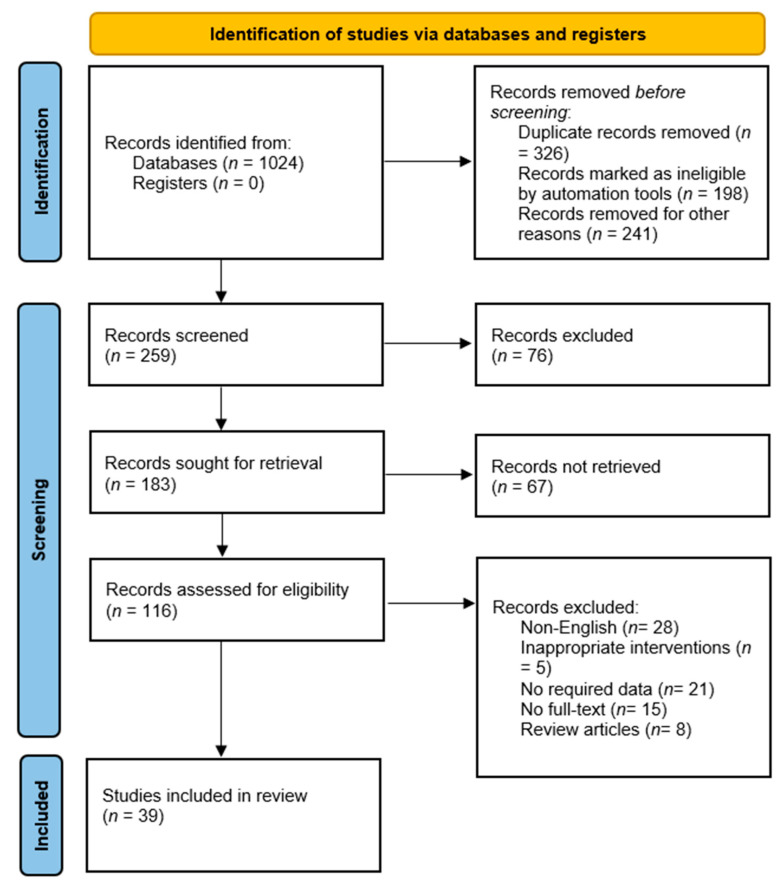
Flowchart of included studies.

The flow diagram in Figure 1 shows that the researchers identified 1024 records from the databases, but only 116 records were assessed for eligibility. At the identification stage, 326 records were excluded due to duplication. During the screening stage, 28 records were excluded because they were not in English. Some records lacked the essential data required for the systematic review. Records that did not have full-text versions available for review were excluded. Review articles, which summarize and analyze existing research, were excluded during the screening stage. After completing the identification and screening stages, the research team identified 39 studies that met the inclusion criteria and were relevant to the systematic review. These studies formed the basis for the subsequent data extraction and analysis, contributing to the comprehensive evaluation of AI systems for early lung cancer detection in the systematic review.

In Table 1, we present an overview of the characteristics of the included studies in our systematic review, each focusing on the application of AI models for the early detection of lung cancer. The table encompasses a diverse range of studies conducted across different countries and utilizing various AI models and data sources. When comparing and contrasting the results of these studies, several key insights emerge. While studies such as Wu et al. (2022) and Alexander et al. (2020) achieved notably high specificity levels, suggesting the potential for reducing false positives in clinical settings, Baldwin et al. (2020) achieved exceptionally high sensitivity, minimizing the risk of missing cancer cases [4,7,10]. On the other hand, the study by Chen (2022) showcases the effectiveness of AI models, specifically CNN and RNN, in improving the overall accuracy of lung cancer prediction [8]. Notably, Huang et al. (2018) integrated sensor array technology with machine learning, demonstrating its promise in the precise identification of lung cancer, especially when compared to traditional models [11]. Li et al. (2019) conducted a retrospective study in China using 3D deep learning technology on CT scans [9]. Their AI system achieved a sensitivity of 75% and specificity of 82%, resulting in an overall accuracy of 88.8%. This research emphasized the potential of AI as a diagnostic tool capable of providing more precise and unbiased outcomes in the diagnosis of pulmonary nodules, ultimately reducing the interpretation time for radiologists. Choi et al. (2018), from the USA, conducted a retrospective study using Support Vector Machine (SVM) and LASSO on LIDC-IDRI data [5]. Their AI model achieved an accuracy of 84.6%, which was notably 12.4% higher than the accuracy for Lung-RADS. This result demonstrated the potential of AI in substantially improving the accuracy of lung cancer detection. In another study from China employed a 3D CMixNet model on LUNA-16 and LIDC-IDRI datasets. Their system achieved a sensitivity of 94.0% and specificity of 91.0%, showcasing better results compared to existing methods for lung cancer detection. These variations in results highlight the trade-offs between sensitivity and specificity, as well as the distinct strengths of different AI models and approaches. While some studies emphasize the potential of AI in overcoming specific challenges, such as PD-L1 assessment or eligibility assessment, others underscore the efficiency and reliability of AI in lung cancer screening. Collectively, these findings underscore the transformative potential of AI in enhancing the accuracy and efficiency of lung cancer diagnosis, promising significant benefits to both patients and healthcare professionals. Collectively, these results underscore the transformative role AI can play in improving the accuracy, efficiency, and reliability of lung cancer diagnosis, ultimately benefiting patients and healthcare professionals. Our systematic review incorporates these findings to offer a holistic understanding of the state of AI in lung cancer detection, shedding light on the remarkable potential of these technologies in the field of oncology.

**Figure 2:**
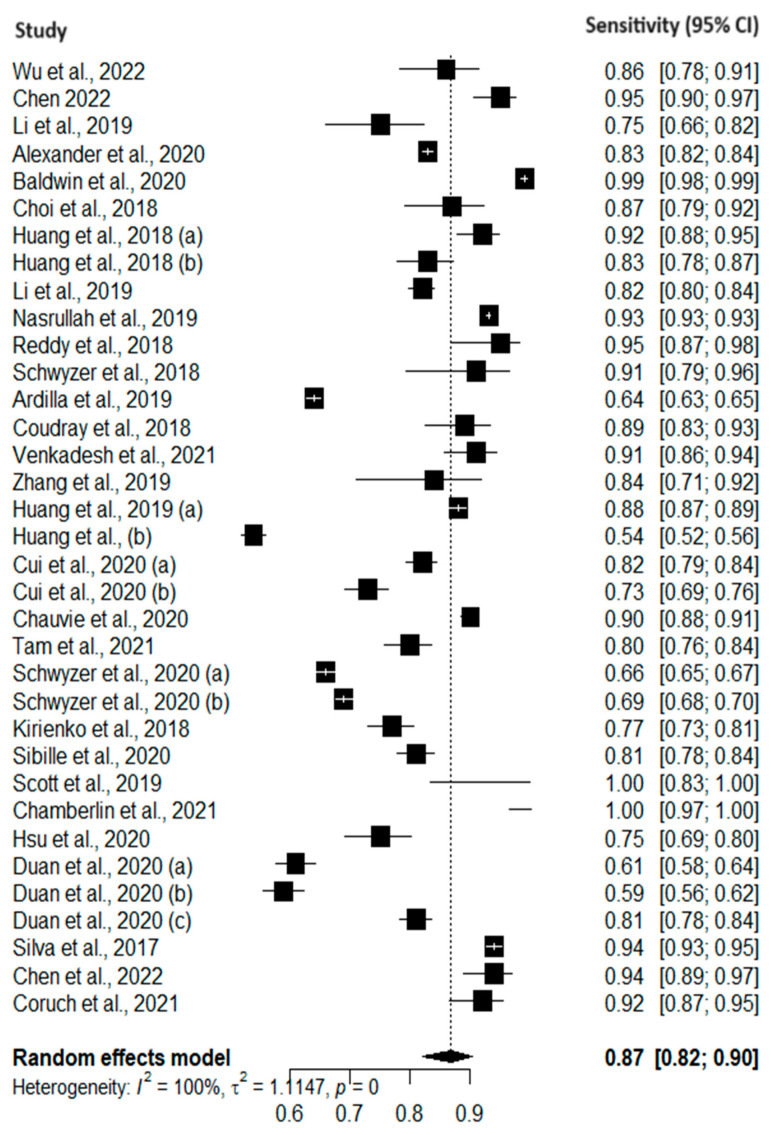
Forest plot of pooled sensitivity of AI models [4,5,7,8,9,10,11,12,13,14,15,16,17,18,19,20,21,22,23,24,25,26,27,28,29,30,31,32,33,34,35,36,37,38,39,40,41,42].

**Figure 3:**
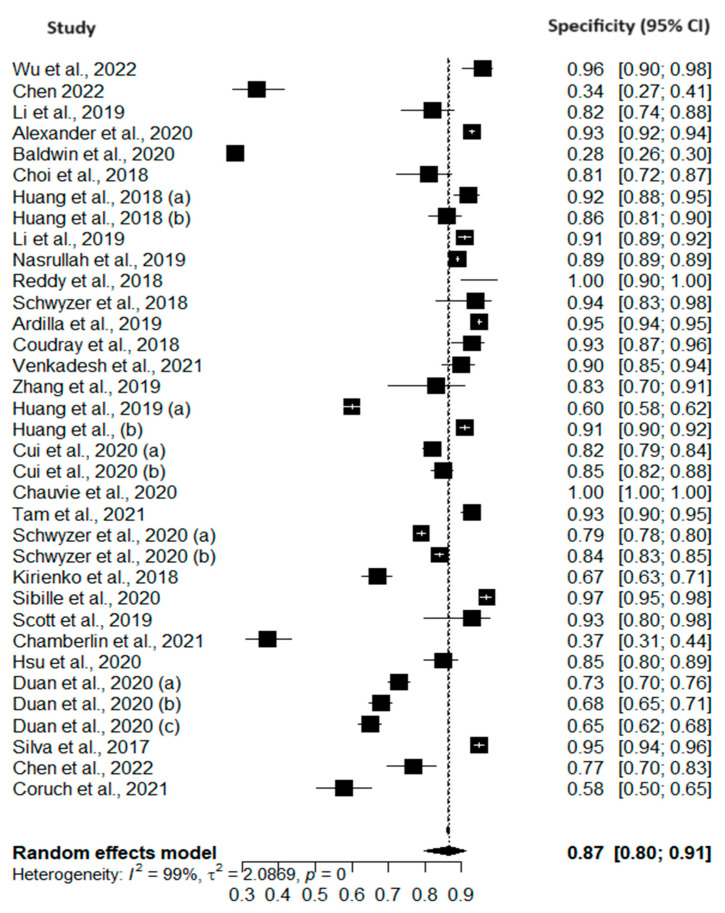
Forest plot of pooled specificity of AI models [4,5,7,8,9,10,11,12,13,14,15,16,17,18,19,20,21,22,23,24,25,26,27,28,29,30,31,32,33,34,35,36,37,38,39,40,41,42].

**Figure 4:**
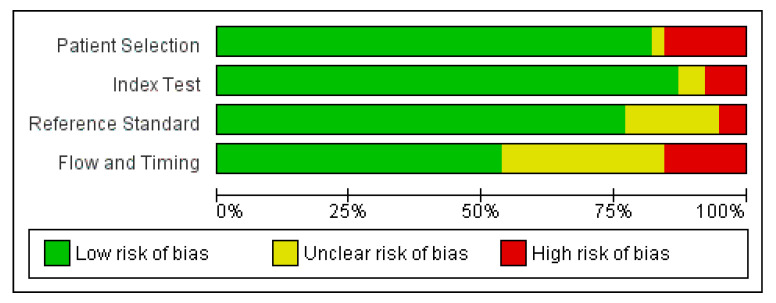
Quality assessment of included studies using QUADAS-2 tool [43].

Figure 2 and Figure 3 present forest plots of the pooled sensitivity and sensitivity of AI models for the early diagnosis of lung cancer. The pooled sensitivity and specificity of AI models across the included studies were 0.87 (95% CI: 0.82–0.90) and 0.87 (95% CI: 0.80–0.91), respectively. These results indicate that AI models demonstrated a high level of accuracy in correctly identifying true positives and true negatives, showing promising results for the early diagnosis of lung cancer. However, heterogeneity was observed among the included studies. This heterogeneity may be attributed to variations in study populations, data sources, and model specifications. The results of quality assessments are presented in Figure 4.

## 4. Discussion

Lung cancer is one of the most prevalent diseases worldwide and the leading cause of cancer-associated deaths, with an estimated 2.2 million new cases and 1.8 million deaths in 2020 [44]. Currently, a CT scan of the chest is the most frequent method of lung cancer screening. Its high resolution can elucidate the association among surrounding organs and blood vessels more clearly, and it plays a significant role in the early detection of lung cancer [45]. However, the accuracy of this method can be influenced by benign lesions such as necrosis, inflammation, tuberculosis, various textures in lung images, and several other factors like the experience of radiologists, potentially leading to misdiagnosis and omissions [46]. With the implementation of AI-assisted diagnostic systems into clinical practice, a new era has dawned in the field of lung cancer diagnosis. Recent studies have documented the growing and widespread utilization of AI models in clinical diagnosis and treatment, respectively [47,48,49]. AI models primarily focus on diagnosing and evaluating various medical images, including skin lesions, pathological microscopic images, and radiological data. AI models are remarkable in their ability to enhance diagnostic accuracy, stability, and work efficiency.

This review documented promising results, indicating that AI models for the early diagnosis of lung cancer demonstrated a high level of accuracy, with pooled sensitivity and specificity values of 0.87 (95% CI: 0.82–0.90) and 0.87 (95% CI: 0.80–0.91), respectively. These findings suggest that AI models exhibit significant capability in correctly identifying true positives and true negatives. Liu et al. recently conducted a systematic review and meta-analysis in which they also demonstrated the commendable performance of AI models in predicting lung cancer, with a pooled sensitivity and specificity of 89% and 87%, respectively [46]. Robust performance is sometimes crucial in terms of lung cancer, where early detections substantially impact patient outcomes. High accuracy of more than 90% was observed in this study, which aligns with the broader trend in the medical literature supporting the effectiveness of AI in diagnostic settings [7,8,10,12,14,15,26,32]. However, a lower pooled accuracy was also reported in studies mainly focused on lung cancer screening, specifically considering the results obtained across all studies [20,30,34,42] and those in which a CNN model was employed [20,30,42], ranging from 67–75%, respectively. Despite these consolidated findings, we are confident that AI models are a valuable resource for radiologists to detect lung cancer.

AI-assisted diagnostic systems result in different diagnostic outcomes. A study reported that a 3D CNN model exhibited greater advantages in detecting lung cancer as compared to improvements seen with other AI models [18]. However, in two studies, ANN achieved high diagnostic performance that could be useful for the detection of lung cancer [32,33]. Distinct algorithms demonstrate diverse diagnostic capabilities, notably in radionics and deep learning, which not only assist in predicting the benign or malignant nature of lung nodules, but also identify the prognosis of small-cell lung cancer [50,51]. The utilization of AI models in clinical practice is promising; however, validity remains a critical step for generalizability. Among 39 articles, 15 articles performed cross-validation to assess the effectiveness and reliability of AI models [5,6,12,14,15,19,21,23,26,28,29,30,36,42,50].

Despite the overall positive outcomes, observed heterogeneity among the included studies was identified. Therefore, future research should focus on refining AI models, considering the identified heterogeneity challenges. Collaborative efforts among researchers, clinicians, and policymakers are essential to establish guidelines and standards for the development and evaluation of AI systems in lung cancer screening. By addressing these challenges collectively, the field can progress toward the implementation of AI technologies in clinical settings, ultimately improving the early diagnosis and management of lung cancer.

The current study has certain limitations that should be addressed: (1) The exclusion of the studies lacking complete diagnostic data may have altered the results. (2) While conducting this comprehensive search, only English language articles were included, potentially introducing language bias. (3) The high heterogeneity among all included studies may be attributed to variations in study populations, data sources, and model specifications, and these results warrant further investigation. (4) The included studies were mainly designed retrospectively, which may have affected the overall quality of the systematic review and meta-analysis. Despite the initial verification of AI models’ effectiveness in lung cancer screening, most of the AI-based approaches are still in the laboratory research stage and have not yet been implemented into clinical practice. Limitations are evident in data integration, image data quality, legal liability definition, complex pathology diagnosis and cost of use. However, a huge volume of experienced healthcare professionals, especially radiologists and pathologists, are getting involved in AI-assisted lung cancer detection. It is anticipated that AI models will play a significant role in the early detection of lung cancer.

## 5. Conclusions

This systematic review and meta-analysis reported the promising outcomes of AI models in the early detection of lung cancer. The pooled sensitivity and specificity values of 0.87 (95% CI: 0.82–0.90) and 0.87 (95% CI: 0.80–0.91) showed the potential of AI models in identifying true positives and true negatives. Regarding the observed heterogeneity among the included studies, these findings highlight the need for standardized protocols in the development of AI models for lung cancer screening. As the medical field continues to grow, healthcare professionals and patients will benefit from the integration of AI models in clinical practice once these models have been validated in large-scale prospective studies.

## Figures and Tables

**Table 1:** Characteristics of included studies.

Author and Year	Country	Study Design	AI Models Used	Source of Dataset	Key Performance Metrics of AI Models			Validation Method	Outcomes
					Sensitivity	Specificity	Accuracy		
Wu et al., 2022 [7]	China	Retrospective study	DL	Whole slide images	86%	96.4%	93.2%	NA	AI-assisted system can be an effective and valuable tool to overcome the challenges of PD-L1 assessment.
Chen 2022 [8]	China	Retrospective study	CNN and RNN	H-E stained pathological slices	95.1%	34.4%	92%	NA	AI-based approach using CNN and RCN could be very effective for improving the accuracy of predicting lung cancer.
Li et al., 2019 [9]	China	Retrospective study	3D deep learning technology	CT scans	75%	82%	88.8%	Internal validation	AI may represent a valuable diagnostic tool that shows more precise and unbiased outcomes in the diagnosis of pulmonary nodules, thus minimizing the time required for interpretation of results by radiologists.
Alexander et al., 2020 [10]	Australia	Retrospective study	ML and NLP	TMC	83.3%	93.8%	91.6%	Internal validation	The AI-assisted system enables efficient and reliable screening of cancer patients, with an accuracy of 91.6% for overall eligibility assessment.
Baldwin et al., 2020 [4]	UK	Retrospective study	LCP-CNN	EDC	99.5%	28%	-	External validation	The LCP-CNN approach minimizes the risk of missing cancer cases when compared to the Brock model.
Choi et al., 2018 [5]	USA	Retrospective study	SVM-LASSO	LIDC-IDRI	87.2%	81.2%	84.6%	Cross-validation	The proposed AI model achieved 84.6% accuracy, which was 12.4% higher than accuracy for Lung-RADS.
Huang et al., 2018 [11]	Taiwan	Prospective study	ML	-	For internal validation 92.3% For external validation 83.3%	For internal validation 92.9% For external validation 86.2%	For internal validation 92.7% For external validation 85.4%	External validation/ internal validation	The integration of the sensor array technique and machine learning enables the precise identification of lung cancer with high accuracy.
Li et al., 2019 [12]	China	Retrospective study	3D deep CNN (SS-OLHF)	LIDC_IDRI	82.6%	91.3%	93.0%	Cross-validation	The proposed fusion algorithm achieved the highest specificity, sensitivity, and accuracy scores among all classification models.
Nasrullah et al., 2019 [13]	China	Retrospective study	3D CMixNet	LUNA-16 and LIDC-IDRI	94.0%	91.0%	-	-	The proposed system, evaluated on LIDC-IDRI datasets, showed better results compared to the existing methods.
Reddy et al., 2018 [14]	India	Retrospective study	GLCM	Microarray data clustering mechanisms	95.3%	100%	97.6%	Cross-validation	GLCM features predicted lung tumor with higher accuracy than histogram features.
Schwyzer et al., 2018 [15]	Switzerland	Retrospective study	ANN	Internal	93.6%	95.5%	94.7%	Cross-validation	ML algorithms may help in fully automated lung cancer detection even at a low effective radiation doses.
Ardila et al., 2019 [16]	USA	Retrospective study	3D CNN	LUNA, LIDC and NLST	64.7%	95.2%	-	Internal validation	DL models have the potential to increase consistency and accuracy and enable the adoption of lung cancer screening.
Coudray et al., 2018 [17]	USA	Retrospective study	CNN	TCGA	89%	93%	-	Internal validation	The outcomes suggest that DL can assist healthcare professionals in the detection of cancer.
Hussein et al., 2017 [18]	USA	-	3D CNN	LIDC-IDRI	-	-	91.2%	Internal validation	The proposed approach achieved cutting-edge outcomes in regressing malignancy scores.
Venkadesh et al., 2021 [19]	Denmark	Retrospective study	DL	NLST and DLCST	For subset A: 91% For subset B: 54%	90%	-	Cross-validation	This algorithm has the potential to provide reliable and reproducible malignancy risk scores for experts, which could contribute to promoting the effectiveness of lung cancer screening management.
Ciompi et al., 2017 [20]	The Netherlands	Retrospective study	CNN	MILD and DLCST	-	-	72.9%	Internal validation	The performance of the proposed DL model in classifying nodule type surpassed that of conventional machine learning approaches.
Petousis et al., 2016 [21]	USA	Retrospective study	DBN	NLST	-	-	-	Cross-validation	The lung cancer screening DBNs were reported to have high discrimination and predictive power with most of the cancer as well as non-cancer cases.
Zhang et al., 2019 [22]	China	Retrospective study	3D CNN	LUNA and Kaggle	84.4%	83%	-	Cross-validation	The 3D CNN with a DL algorithm may assist experts by providing accurate data for diagnosing pulmonary nodules.
Petousis et al., 2019 [23]	USA	Retrospective study	ML and DBN	NLST	-	-	-	Cross-validation	The proposed model reduced the FPR while maintaining TPR, and improved early prediction of cancer cases.
Huang et al., 2019 [24]	USA	Retrospective study	DL	NLST and PanCan	88%	60%	-	External validation	DL scores could be used for early detection of cancer cases.
Cui et al., 2020 [25]	China	Retrospective study	DL	LUNA	90%	85%	-	External validation	The DL system had better identification sensitivity and performance than that of the experts.
Chauvie et al., 2020 [26]	Italy	Retrospective study	Neural network	-	90%	100%	100%	Cross-validation	The utilization of visual analysis along with NN could help experts to reduce the number of false positive cases.
Tam et al., 2021 [27]	UK	Retrospective study	DNN	NHS Cancer Registry Database	80%	93%	87%	-	The proposed AI resulted in a reduction in radiologist errors and improved clinician reporting performance.
Schwyzer et al., 2020 [28]	Switzerland	Retrospective study	DL	-	For BSREM 69.2% For OSEM 66.7%	For BSREM 84.5% For OSEM 79.0%	-	Cross-validation	AI performed substantially better on images with BSREM than OSEM.
Teramoto et al., 2010 [29]	Japan	Retrospective study	CNN	-	90.1%	-	-	Cross-validation	CNN technique can be very effective for the early detection of pulmonary nodules in PET/CT images.
Kirienko et al., 2018 [30]	Italy	Retrospective study	CNN	Independent dataset	-	67%	69%	Cross-validation	CNNs can be used as a reliable tool to assist in the staging of lung cancer patients.
Sibille et al., 2020 [31]	US and Germany	Retrospective study	Deep CNN	Independent dataset	81.0%	97.3%	-	Independent internal validation	CNN achieved high diagnostic performance when both PET and CT images were utilized.
Toney et al., 2014 [32]	USA	Prospective study	ANN	Independent dataset	-	-	99.2%	-	ANNs can provide more accurate and consistent assessment of nodal stage in lung cancer patients.
Scott et al., 2019 [33]	USA	Retrospective study	ANN	Independent dataset	100%	93.1%	-	External validation	ANNs have potential to improve diagnostic certainty and can be useful to help direct clinical and imaging follow-up.
Hyun et al., 2019 [34]	Korea	Retrospective study	RF, NN, NBS, LL, SVM	Independent dataset	SVM 52.6% RF 52.6% NN 52.6% NBS 52.6% LL 52.6%	-	SVM 67.1% RF 67.1% NN 67.1% NBS 67.1% LL 67.1%	Internal validation	An ML approach with PET-based radiomics aids in early detection of lung cancer.
Jayasurya et al., 2010 [35]	Belgium and the Netherlands	Retrospective study	BN	Independent dataset	-	-	-	External validation	BN models are better at handling missing data as compared to SVM models and are more suitable for the medical domain.
Luo et al., 2018 [36]	USA	Retrospective study	BN	Independent dataset	-	-	-	Cross-validation	The proposed BN model is stable and can identify hierarchical relationships among biophysical features for the prediction of lung cancer.
Chamberlin et al., 2021 [37]	USA, Europe, and Asia	Retrospective study	CNN	Independent data set	100%	70.8%	-	Internal validation	AI software strongly agrees with expert radiologist determination of detection of both lung nodules and CACV.
Hsu et al., 2020 [6]	Taiwan	Retrospective study	ANN	Hospital-based cancer registry	75.0%	85.0%	-	Cross-validation	This study reported that ANN had better sensitivity for the detection of lung cancer than Lung-RADS.
Duan et al., 2020 [38]	China	Retrospective study	Decision tree C5.0, ANN, SVM	-	C5.0-1 61.8% SVM 59.2% ANN 81.5%	C5.0-1 73.4% SVM 68.7% ANN 65.6%	-	-	These AI models can be utilized for the on-site screening and clinical diagnosis of the high-risk population.
Silva et al., 2017 [39]	Brazil	Retrospective study	Deep CNN	LIDC-IDRI	94.6%	95.1%	94.7%	Internal validation	The proposed DL models demonstrated promising performance and avoided the need for feature extraction and selection steps.
Trajanovski et al., 2021 [40]	USA	Retrospective study	CNN	Kaggle, NLST, and UCM	93%	-	-	External validation	The proposed DL had a better sensitivity of 93% and can be used for the screening of lung cancer.
Chen et al., 2022 [41]	China	Retrospective study	CNN	Self-built data from hospital	94.1%	77.7%	87.1%	External validation	The DL-based AI film reading system has higher sensitivity for the diagnosis of NSCLC than radiologists.
Coruch et al., 2021 [42]	Turkey	Retrospective study	CNN	Hospital records	92.2%	58.7%	75.2%	Cross-validation	The performance of the observers in evaluating the risk of malignancy was slightly higher than the performance of fusion AI algorithms.

PD-L1 = programmed death-ligand 1, CNN = convolutional neural network, RNN = recurrent neural network, ML = machine learning, NLP = natural language processing, TMC = thoracic malignancies cohort, SVM-LASSO = support vector machine–least absolute linkage and selection operator, EDC = electronic data capture, RADS = Reporting and Data System, CMixNet = customized mixed link network, LIDC = lung image database consortium, IDRI = image database resource initiative. GLCM = gray level co-event matrix, NLST = National Lung Cancer Screening Trial, LUNA = lung nodule analysis, TCGA = the Cancer Genome Atlas, DLCST = Danish lung cancer screening trial, MILD = multicenter Italian lung detection, RF = random Forest, BN = Bayesian network, DNN = deep neural network, BSREM = block-sequential regularized expectation maximization, OSEM = ordered subset expectation maximization, ANN = artificial neural network, GGO = ground-glass opacities, LL = logistic regression, NBS = naïve Bayesian system, NN = neural network, UCM = University of Chicago.
